# The Status of Facility Based Emergency Care in Public Hospitals of Ethiopia Using WHO Assessment Tool

**DOI:** 10.4314/ejhs.v32i6.5

**Published:** 2022-11

**Authors:** Menbeu Sultan, Woldesenbet Waganew, Lemlem Beza, Yemene GebreMedihin, Mulu Kidane

**Affiliations:** 1 Saint Paul's Hospital Millennium Medical College, Department of Emergency and Critical Care, Addis Ababa, Ethiopia; 2 Addis Ababa Univercity department of emergency medicine

**Keywords:** Emergency Care, Emergency department

## Abstract

**Background:**

The availability of emergency care contributes to half of the total mortality burden in a low and middle income countries. The significant proportion of emergency departments in LMICs are understaffed and poorly equipped. The purpose of this study is to examine the status of emergency units and to describe the facilitators and barriers to the provision of facility-based emergency care at selected Ethiopian public hospitals.

**Methods:**

A mixed-methods explanatory design was used. Ten hospitals were purposively selected due to their high number of patients and referral service. A WHO facility assessment tool was used to quantitatively assess the facilities, and an in-depth interview with hospital and emergency room leadership was conducted. The quantitative results were descriptively analyzed, and the qualitative data was thematically analyzed.

**Result:**

This survey included a total of ten hospitals. Three of the facilities were general hospitals, and seven were tertiary level hospitals. They all were equipped with an emergency room. All of the studied hospitals serve a population of over one million people. In terms of infrastructure, only 3/10 (30%) have adequate water supply, and alf (5/10) have telephone access in their ED. The qualitative resultshowedthat the most common barriers to emergency care delivery were prolonged patient stays in the emergency room, inadequate equipment, and a shortage of trained professionals.

**Conclusion:**

The status of emergency care in Ethiopia is still developing, and hospital care as a whole should improve to alleviate the high burden of care in emergency rooms and reduce morbidity and mortality.

## Introduction

Ethiopia with over 116 million current populations, it is the 12^th^ most populous country in the world and the second in Africa after Nigeria(1). The country has a rapidly growing population, which is almost expected to double by 2050 and has a median age of 19.5 years. The GDP per capita was $542 in 2017, and the all-time highest GDP per capita of $602.20 in 2019 puts the country in the World Bank low-income countries' category (2–4). The country has less developed and organized emergency medical care system (5).

Emergency care for all acutely ill and injured patients is a crucial component of a functioning health system and is required for universal health coverage(UHC)to be implemented. According to WHO estimates 2020, illnesses that can be treated with emergency care account for 54% of deaths in low- and middle-income countries(6). The emergency departments (EDs) care demand is affected by different factors that include social, epidemiological and features related to the organization of the health system (7). In the presence of poor service organization, there's a possibility that both the quality and safety of care will be compromised, especially if capacity can't keep up with demand (8). Despite the fact that emergency medicine is a relatively new discipline, Ethiopia has achieved significant progress in developing a robust emergency care system in a relatively short period of time (8).

Ethiopia has one of the highest mortality burdens from emergency conditions. It accounts 1154 per 100,000 people and death and disability-adjusted life years of 47,728 per 100,000 people.Consecutively, the country has one of the lowest national emergency usage rates of 8 per 1000 people(9). This, one of the lowest emergency usage rates, could be due to several barriers; however, there is not enough evidence to support this facts. The status and demand of emergency care depends on the sustainable support and health care system organization. In addition, the =repeated conflicts, pandemic, and epidemic disease may have affected its status (10)(11). However, there is a shortage of published evidence on the barriers and facilitators of emergency care provision in Ethiopia. Hence, this study aimed to assess the status of facility-based emergency service in selected hospitals and explore the facilitators and barriers to the provision of emergency care in Ethiopia.

## Methods

**Research Design**: An explanatory mixed-methods study design was used in this study. A cross-sectional assessment of 10 purposively selected public hospitals was performed using the WHO Emergency Unit Assessment Tool (HEAT)(12). In-depth interview was conducted withleaders from emergency departments and hospitals.

**Study setting**: The study was carried out in 10 public hospitals emergency roomsin Ethiopia. The hospitals were situated in the country's major cities. The hospitals were chosen based on their capacity as tertiary hospitals, the proportion of patients and referral services.

**Data collection and tools**: The WHO HEAT data collection tool was used to collect data in English. HEAT variables include facility characteristics, human resources, clinical services, signal functions, and essential resources for emergency care. Emergency physicians and emergency and critical care nurses collected the data. The research team developed an in-depth interview guide, which was used to interview hospital and emergency room leaders. In addition to the interview guide, the preliminary result from qualitative study was used as interview guide to identify the facilitators and barriers for emergency room care delivery. Each interview lasted 25 to 40 minutes.

**Data analysis**: Descriptive data analysis was done using Stata version 17.1 software, and the qualitative data was organized thematically using open code. The hospital's medical director, emergency room directors, and a senior nurse were among those interviewed. The qualitative data was audio recorded and translated. Content was analyzed using inductive thematic analysis and the outcome was organized thematically.

**Ethical clearance**: The ethical principles outlined in the Declaration of Helsinki guide the entire research process.Ethical approval was secured from the Research and Ethical Review Board of Saint Paul's Hospital Millennium Medical College after obtaining permission letter was from the Ministry of Health.

## Results

**Quantitative result**: Three of the ten hospitals involved were general hospitals, with the rest being tertiary care facilities. All the involved hospitalshad a dedicated emergency room with 11 to 60 bedsand an ICU bed, ranging from 4 to 16 beds. The majority of the hospitals involved provided over a million people and had a substantial proportion of outpatient visits. Details of the hospitals characteristics is shown in Table 1 below.

**Table 1:** General characteristics of selected hospitals, Ethiopia 2022

Hospital name	Address	Number of community served	Emergency unit visit per year	Outpatient visit Per year	Bed for emergency	Resuscitation beds	Inpatient- hospital beds	ICU beds	Presence EM physician
HA2	Addis Ababa	7–10 million	8960	480000	60	3	136	14	Yes
HJ1	Jimma	6 million	23202	780000		4	141	12	Yes
HB1	Bahidar dar	7Million	11214	400000	11	4	280	6	No
K2HTB1	Addis Ababa	1.2million	8600	480000	45	6	298	6	Yes
HK1	Jigjiga	1million	15000	42546	19	6	350	8	No
DH1	Afar region	1.2million	16117	72540	26	7	448	6	No
HH1	Hawassa	6million	7770	630000	48	8	500	9	Yes
ZM1	Addis Ababa	1million	8100	ND	51	8	500	8	Yes
HA!	Addis Ababa	ND	11713	110000	14	2	100	16	Yes
HF1	Harar	1million	20000	130044	30	9	790	4	Yes

**ED infrastructure and essential equipment**: As illustrated in Table 2, only 3/10 (30%) of the hospital emergency departments studied had adequate water supply, half (5/10) had telephone access in their ED, and only 2/10 (20%) had electronic documentation. In terms of emergency room zones, 80% had a triage area, and staff were only 20% and 10%, respectively. There was a scarcity of emergency equipment and supplies.40 % had a waiting area, and 60 percent had an emergency pharmacy. The availability of toilets for patients.

**Table 2:** Infrastructure and essential equipment availability in emergency units

Item	Generally unavailable n(%)	Some availability n(%)	Adequate n (%)
Water supplies	1(10)	6(60)	3 (30)
Electricity source generator/wire	1(10)	1(20)	7(70)
Telephone	1(10)	4(40)	5(50)
Easy physical access for patient with stretcher /wheelchair	-	2(20)	8(80)
Documentation			
Paper based	1(10)	2(20)	7(70)
Electronic	6(60)	2(20)	2(20)
Zones in the emergency room			
Waiting area	3(30)	3(30)	4(40)
Triage area	-	2(20)	8(80)
Resuscitation	1(10)	4(40)	5(50)
Isolation area	1(10)	3(30)	6(60)
Emergency pharmacy	2(20)	2(20)	6(60)
Staff dedicated working area	-	4(40)	6(60)
Store	-	4	6
Toilet accessibility			
Patient	2	6	2
Staff	4	5	1
Equipment and supplies			
PPIs	-	6	4
Cardiac monitor	1	5	4
ECG machine	1	3	6
Crash cart with high acuity equipment	2	3	5
Oxygen sources	Yes	No	
Concentrator	7	3	
Tank	9	1	
Pipe	1	9	

**Laboratory and imaging facilities**: Nine out of the ten hospitals were conducting emergency-related laboratories. As illustrated in Table 3 below, nine of them performed CBCs, electrolytes, and many other tests, but only one institution had ABG. Cardiovascular and pancreatic biomarkers are only performed in half of the hospitals. Only two out of ten hospitals had mobile x-rays. In case of emergency, every hospital had a portable ultrasound.

**Table 3:** laboratory and imaging availability in emergency units 2022

Laboratory service	Yes (%)	No(%)
CBC	9(90)	1(10)
Hgb	9(90)	1(10)
Coagulation profile	7(70)	3(30)
Electrolyte	9(90)	1(10)
BUN/Creatinine	9(90)	1(10)
Lipase	5(50)	5(50)
Cardiac troponin	5(50)	5(50)
ABG	1(10)	9(90)
Cross match	9(90)	1(10)
Blood culture	6(60)	4(40)
System to report critical /urgent laboratory result	5(50)	5(50)
Rapid HIV test	8(80)	2(20)
Pregnancy test	8(80)	2(20)
Blood glucose	10(100)	-
Urine dipstick	8(80)	2(20)
Imaging service X-ray (hospital)	8(80)	2(20)
Portable X-ray in the ER	2(20)	8(80)
Ultrasound (ER)	10(100)	-
CT scan (hospital)	6(60)	4(10)
System to report critical imaging result	7(70)	3(30)

**Availability of protocols and guide line**: The percentage of emergency centers which are utilizing protocol and guidelines were very low in the surveyed hospitals. As table 2 belowshows, all hospitals had triage protocols but case management protocols availabilitywaslow. Protocol for acutely ill patient management accounted 3/10 (30%), fluid resuscitation account 60%. Protocol for hand - over and referral protocols accounted 30% and 40 %, respectively.

**Qualitativ findings:**

On the facilitators and barriers to the delivery of emergency care in hospitals, interviews with emergencyunit and hospitals leadership were conducted.In addition, to the interview guide, the preliminary quantitative result were used to frame the discussion. Throughout the analysis, three themes came into focus which were barriers and facilitators to emergency unit care delivery in the facilities. These recurring themes include human resources, infrastructure constraints, and system-related concerns.

**System related factors**: The hospital leadership agreed that improved emergency care was a facilitator for delivery of emergency care, but the barriers were an underdeveloped tertiary care system, including ICU care and imaging, which delayed patient disposition, leading to overcrowded emergency rooms.

*“The reason for prolonged stay in the emergency units were bed unavailability in the wards, investigation delay, unsettled diagnosis, and delayed consultation”.SP1*

Under developed subspecialty care and difficulty in interfaculty transfers were also mentioned as a barrier for poor emergency care delivery.

*“Our hospital do not have much subspecialty care. For instance we do not provide orthopedic surgical service, hence patients wait in the emergency room for long time until they find bed in other hospital.” ZM1*

*“Patients are coming to our emergency room with no communication and delayed in the primary care units and when we need referral out, the receiving hospitals may not accept the patient due to space issue”TB2*

*“Regarding the mentioned protocols, most protocols are either difficult for us to develop them or the federal ministry of health do not provide us with an updated protocols.Hence, we rely on physician decision in the management of patients than protocols” SH2*

*“Our emergency room was not built to provide emergency care, we converted the hospital corridor to be an emergency room hence it is not a convenient place to provide an emergency care” TB!*

*“The service demand for emergency careis increasing while we are not improving the infrastructures required for ED, this will hamper the care” AS1*

**Infrastructures**: Key informants discussed the fact that emergency rooms were not adequately equipped to meet the growing demand for emergency care. Some professionals interviewed also stated that emergency care was emerging as a continuum of care. But, the infrastructures starting from pre hospital to intensive care units are not well developed.

*“Our Emergency rooms are providing critical care service with lack of essential equipment and staff training for advanced care also contributing to the ED mortality and crowding.”BLH2*

**Man power**: The discussions also revealed that the growing number of emergency and critical care trained professionals represent an opportunity to improve emergency care; however, The participants raised the existing shortage of emergency trained nurses and physicians, as well as the high turnover of these professionals, which have made emergency room care very difficult.

*“Critically ill patients are staying for a long time in emergencyroom, but the human resources, both nursing and physicians, are not well trained to manage the critically ill patients staying in emergency room for prolonged time” ZM1*

*“Since the remuneration system is poor, they are either unmotivated or want to rotate to other places “JJG1*

*“Consultation service from other departments is very difficult. This happens due to the shortages of specialists and subspecialists in the disciplines. Most patients coming to my emergency units need subspecialty level care but the human resource is inadequate to provide this care” SP1*

## Discussion

The survey done on 10 selected public hospitals providing care to large number of patients showed they had varying number of emergency beds, ranging from 11 to 60 beds, but the number of resuscitation and critical care beds in the hospitals was low. None of the hospital had the recommended number of ICU bed, which is 5 to 20 % of in-patient bed and none of them had the recommended number of resuscitation beds. These hospitals also had limited infrastructure to care for the increasing demand of emergency service in the country. In this regard, 50% of them lackland line telephone access and 40% had no ER pharmacy, which were rate limiting infrastructures.

The observational and qualitative study revealed the emergency unitswere overcrowded with prolonged length of stay. Emergency department overcrowding is a serious problem in many countries. It is a public health crisis that negatively impacts patient safety, worsens the quality of care, and increases mortality(12). Emergency department crowding depends on three determinant variables. These are the number of patients arriving, the time taken to process or treat patients, and the number of patients leaving the ED(13). Emergency department crowding is a multifactorial problemresulting in increased ED waiting times, decreased patient satisfaction and deleterious domino effects on the entire hospital (14). Most of selected hospitals lacked imaging and laboratory service for emergency service; only 20% of hospitals had portable X-ray which could have contributed to the prolonged length of stay and overcrowding in the ED.

In most of Africa countries, formal emergency medicine training started in the last couple of decades. Although it has shown a good progress, emergency medicine is among the youngest specialties in Ethiopia(15)(16). A study from 23 public hospitals in Zambia, a lower middle-income country in Southern Africa, reported lack of training for health care workers as the top barrier to health care delivery followed by shortage of equipment and medications. However, the study concludes that Zambian public hospitals had acceptable capacity to care for emergency conditions despite the reported barriers (17–20). Similarly, another study from the Kingdom of Eswatini, a lower-middle income country, emphasized lack of training, supplies, medications and equipment as limiting factors for performing life-saving procedures (21).

Protocols and guidelines are important to reduce variations in practice and improve quality of care. Guidelines are used to reduce practice variation, guide appropriateness, and measure quality of care. Ultimately, the goal of a guideline is to improve patient outcomes through a change to evidence-based physician practices (22–24). Complete adherence to early goal-directed therapy had shown a significant reduction in 28-day mortality rate, whereas partial adherence had not shown beneficial effect (25, 25). In our study hospitals, the availability of protocols and guidelines were either low or underutilized. Practice guidelines serve as useful tools for clinical decision making.

There is a significant progress in emergency care in Ethiopia. Most of the study hospitals had emergency physician in their emergency units, which indicated the number of emergency and critical care physician and nurses has increased. In addition, there is a focus given by the government to improve the availability of equipment and consumables in emergency.

This study was done using WHO facility emergency unit assessment tool but it does not fit to all settings. There is a need to establish criteria for facility-level emergency care provision, including the introduction of national emergency care policies and Standard Operating Procedures. Stakeholders from seventeen different countries, including Ethiopia, have proposed barriers to effective emergency care as one of the top priority research questions in LMICs along with identification of context-relevant emergency care indicators, accuracy and impact of triage tools, potential quality improvement via registries, characteristics of people seeking emergency care, best practices for staff training and retention, and cost-effectiveness of critical care(22) (23).

This study limitation was, it involved few public hospital where primary care hospitals and private institutions were not included hence generalizability may be difficult. Despite this, it can be used as baseline survey to implement future improvement projects like mentorship of the hospitals, to aware how to standardize emergency room care and better preparation of the emergency units to address the growing demand in emergency care and adherence to basic protocols and procedures.

List of abbreviation/acronyms

## Figures and Tables

**Table 4:** Utilization of protocol and guide line for emergency conditions

Protocols and guide line availability	Yes (%)	No
Triage protocol	10()	0(0)
Acutely ill or injured patient protocol	4(40)	6(60)
Syndromic surveillance guidelines	6(60)	4(40)
Protocol for ED overcrowding communication with hospital	4(40)	6(60)
Protocol for mass causality response	7(70)	3(30)
Protocol for initial ABCD management	6(60)	4(40)
Protocol for medical case management	7(70)	3(30)
Fluid resuscitation protocol	6(60)	4(40)
Protocol for post exposure prophylaxis	6(60)	3(30)
Protocol for timely emergency patient disposition	5(50)	5(50)
Protocol for convening information for patient discharge	4(40)	6(60)
Handover protocol	6(60)	4(40)
Referral/ transfer protocol	3(30)	7(70)
Protocol for managing hazardous exposures (including designated decontamination	7(70)	3(30)
Protocol to protect security of patient, & staff	2(20)	8()80
Trauma registration	5(50)	5(50)
